# The role of molecular biomarkers in recurrent glioblastoma trials: an assessment of the current trial landscape of genome-driven oncology

**DOI:** 10.1007/s12032-024-02501-7

**Published:** 2024-09-24

**Authors:** Mark P. van Opijnen, Filip Y. F. de Vos, Edwin Cuppen, Marjolein Geurts, Sybren L. N. Maas, Marike L. D. Broekman

**Affiliations:** 1 Department of Neurosurgery, Leiden University Medical Center, Albinusdreef 2, 2333 ZA Leiden, the Netherlands; 2 Department of Cell and Chemical Biology, Leiden University Medical Center, Albinusdreef 2, 2333 ZA Leiden, the Netherlands; 3 Department of Medical Oncology, Utrecht University Medical Center, Utrecht, the Netherlands; 4 Hartwig Medical Foundation, Amsterdam, the Netherlands; 5 Center for Molecular Medicine and Oncode Institute, University Medical Center Utrecht, Utrecht, the Netherlands; 6 Departments of Neurology and Medical Oncology, Brain Tumor Center, Erasmus MC Cancer Institute, Rotterdam, the Netherlands; 7 Department of Pathology, Leiden University Medical Center, Leiden, the Netherlands; 8 Department of Pathology, Erasmus MC Cancer Institute, Rotterdam, the Netherlands; 9 Department of Neurosurgery, Haaglanden Medical Center, Lijnbaan 32, 2512 VA The Hague, the Netherlands

**Keywords:** Recurrent glioblastoma, Clinical trial, Molecular testing, Targeted treatment, Genome-driven oncology

## Abstract

For glioblastoma patients, the efficacy-targeted therapy is limited to date. Most of the molecular therapies previously studied are lacking efficacy in this population. More trials are needed to study the actual actionability of biomarkers in (recurrent) glioblastoma. This study aimed to assess the current clinical trial landscape to assess the role of molecular biomarkers in trials on recurrent glioblastoma treatment. The database ClinicalTrials.gov was used to identify not yet completed clinical trials on recurrent glioblastoma in adults. Recruiting studies were assessed to investigate the role of molecular criteria, which were retrieved as detailed as possible. Primary outcome was molecular criteria used as selection criteria for study participation. Next to this, details on moment and method of testing, and targets and drugs studied, were collected. In 76% (181/237) of the included studies, molecular criteria were not included in the study design. Of the remaining 56 studies, at least one specific genomic alteration as selection criterium for study participation was required in 33 (59%) studies. Alterations in *EGFR, CDKN2A/B* or *C, CDK4/6*, and *RB* were most frequently investigated, as were the corresponding drugs abemaciclib and ribociclib. Of the immunotherapies, CAR-T therapies were the most frequently studied therapies. Previously, genomics studies have revealed the presence of potentially actionable alterations in glioblastoma. Our study shows that the potential efficacy of targeted treatment is currently not translated into genome-driven trials in patients with recurrent glioblastoma. An intensification of genome-driven trials might help in providing evidence for (in)efficacy of targeted treatments.

## Introduction

At the inevitable time of glioblastoma recurrence, re-resection, chemotherapy, radiotherapy or combinations of these are still the most commonly used treatment modalities [1–3]. The introduction of targeted therapies and immunotherapies has led to new optimism in other, systemic cancers, although drug resistance and side effects remain challenging drawbacks [4, 5]. New targets and treatments are being investigated, highlighting a continuing interest in precision oncology. In neuro-oncology, however, the success rate of targeted therapy is limited to date [6]. This is largely explained by the fact that the blood–brain barrier and the blood–tumor barrier hamper effective drug delivery and penetration [7, 8]. The *BRAF p.V600E* mutation is currently the only example of an evidence-based target for recurrent glioma, targeted by dabrafenib/trametinib and with response rates around 30% [6, 9]. In patients with isocitrate dehydrogenase (*IDH*) mutant gliomas, the *IDH* inhibitor vorasidenib showed a significant improvement in the progression-free survival [10]. Other molecular therapies previously suggested in neuro-oncology are either tumor agnostic and/or lacking efficacy in brain tumors and are therefore not standard of care [6]. Thus, although several targets have been studied before, there is still a knowledge gap of potentially actionable targets without solid evidence for either efficacy or inefficacy in glioblastoma *IDH* wild-type (IDHwt) patients.

Therefore, this current lack of evidence of the efficacy of genome-driven oncology in glioblastoma patients should not paralyze the exploration of new potentially actionable targets. For instance, hypothetical druggable alterations were found in all but one of the 42 glioblastoma samples analyzed by whole-genome sequencing (WGS) [11]. At the same time, it was shown that the glioblastoma driver instability after standard-of-care primary treatment affects the design of genome-driven trials [12]. Hence, the feasibility of routinely sequencing the whole genome of patients with recurrent glioblastoma in order to maximize targeted treatment options is currently being explored [13].

To better address challenges regarding implementation of genome-driven oncology for patients with glioblastoma, (confirmatory) studies are needed to further study the actionability of biomarkers in this population [1, 6, 14]. This study aimed to assess the current clinical trial landscape to describe the role of genome-driven treatment in the trials on recurrent glioblastoma treatment by picturing the specific potentially actionable targets or systemic therapies that are now being investigated.

## Methods

### Search strategy

A search in the online database of clinical research studies ClinicalTrials.gov was conducted up to June 13, 2024 to identify clinical trials on recurrent glioblastoma in adults. The search terms ‘glioblastoma’ and ‘recurrent’ were combined with filtering on adult patients. No additional filters were applied. This search strategy on ClinicalTrials.gov automatically included other tumor types, which required manual and record by record screening according to the following criteria.

### Selection criteria

This study included all studies on recurrent glioblastoma, primarily based on ClinicalTrials.gov classification and subsequently based on description of the inclusion criteria provided by the investigators. Studies solely on newly diagnosed glioblastoma (in which experimental therapies are not applied) or other tumor types or studies including pediatric patients or medical devices were excluded. Likewise, studies on imaging, radiotherapy, surgery, or anti-cancer diet were also excluded. Diagnostic molecular criteria were not part of the selection criteria. Subsequent selection was based on the current recruitment status: completed, terminated, withdrawn, suspended, or no longer available studies were excluded since details on previously studied molecular targets were beyond the scope of this study. Instead, next to recruiting studies, trials with status ‘available,’ ‘not recruiting,’ or ‘unknown’ were included as well to secure a comprehensive overview of the current and upcoming trial landscape.

### Data extraction

The role of molecular criteria in studies included in the final analysis was assessed by reading the detailed description, eligibility criteria, and study plan (including design and outcome measures) of the study. For those studies with at least one specific genomic alteration as a selection criterium for study participation, details on target(s) and/or drugs studied and moment of molecular diagnostic (i.e. testing on fresh or archival tissue) were then retrieved. Next to this, details on target analysis method (e.g. DNA or RNA sequencing, immunohistochemistry (IHC) or fluorescent in situ hybridization (FISH)), study phase, number of study participants, and recurrence (first or second) were collected.

## Results

### Search results

**Fig. 1:**
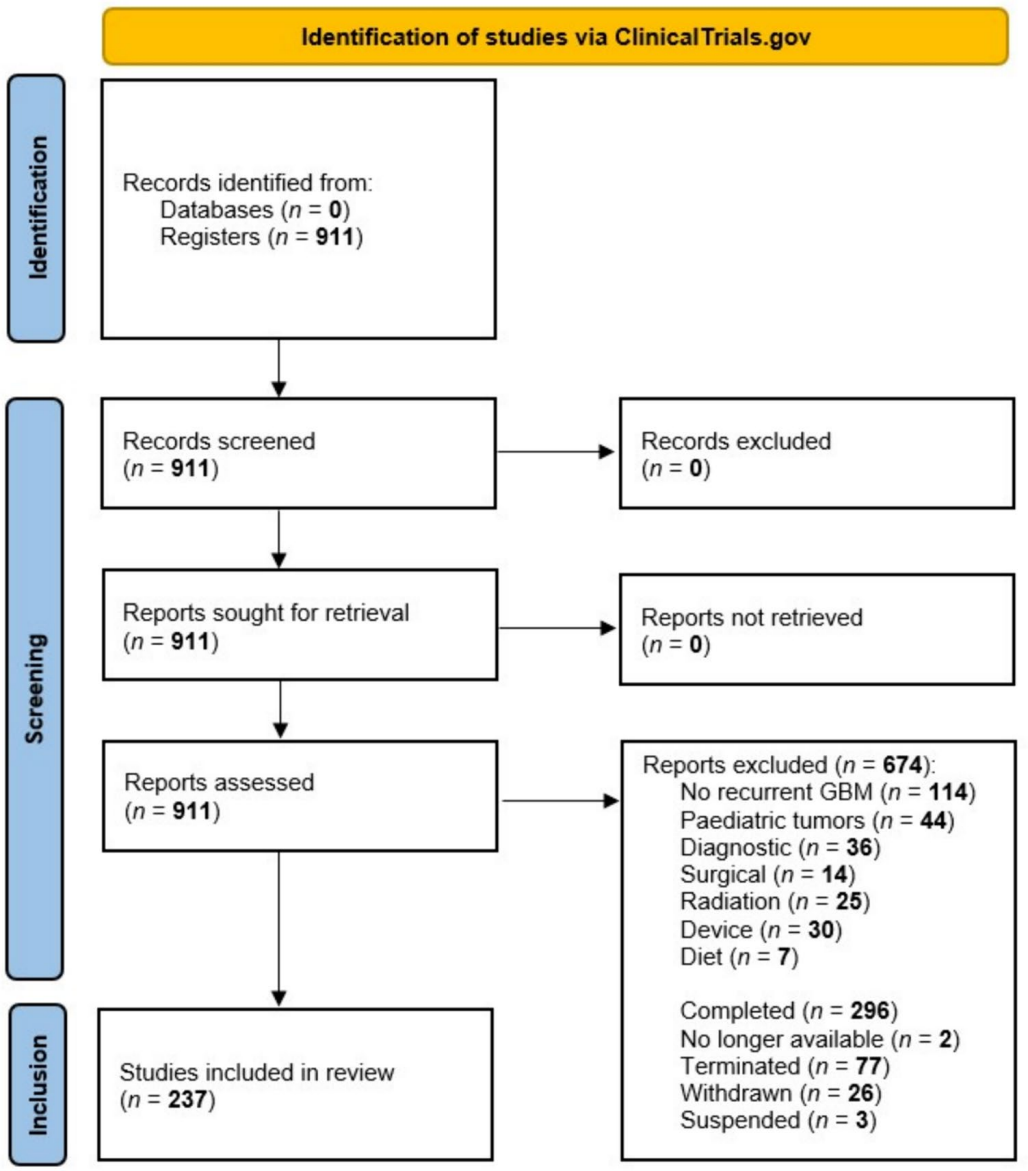
Study selection process. *GBM* glioblastoma

The search strategy resulted in a total of 911 records. Of these, 270 records were excluded based on the objective and/or design of the study. Subsequently, another 404 records were excluded based on the recruitment status of the study. As a result, a total of 237 records were classified eligible and included for molecular criteria assessment. See Fig. 1 for an overview of the selection process.

### Study characteristics

In 181 (76%) of the 237 included studies, molecular criteria (other than diagnostic) were not included in the study design. Of the remaining 56 studies, at least one specific genomic alteration as an upfront inclusion criterium for study participation was required in 33 (59%) of those studies (Table 1). The remaining 23 (41%) studies applied molecular criteria after patient inclusion, for instance, for drug response correlation. The mean number of study participants in these 33 studies was 38 (range 10–200). The most frequent study phase was 1 (64%, 21/33), followed by phase 2 (24%, 8/33) and phase 1–2 (12%, 4/33). Looking to the in-/exclusion criteria, in most of these studies the glioblastoma recurrence was not specified (73%, 24/33), but was occasionally limited to first (21%, 7/33) or ‘first or second’ (6%, 2/33) recurrence. The requirement that molecular testing was performed on fresh tumor material (i.e. at recurrence) was not provided in most studies. In two studies, fresh material was used (6%, 2/33), while in 8 studies archival (i.e. from primary setting) and/or fresh tissue was used for molecular testing (24%, 8/33). In the remaining studies either archival tissue sufficed (30%, 10/33) or a requirement regarding the moment of molecular testing was not provided (39%, 13/33). Testing was done by next-generation sequencing (NGS) or RNA sequencing (RNAseq) in 8 and 1 of the 33 studies, respectively. IHC, FISH, and sequencing of DNA via tumor in situ fluid (TISF) collection were used in 14, 3, and 1 of the studies, respectively, while the target analysis method was not specified in 11 of the 33 (33%) studies.

**Table 1:** Details of studies including molecular criteria in the study design

Category	Number (%)
Studies with specific variant(s) as inclusion criterium (*n* = 33)	
Genes	
* EGFR*	11 (33%)
* CDK4/6*	4 (12%)
* CDKN2A/B/C*	4 (12%)
* RB*	4 (12%)
* HER2*	3 (9%)
*PTEN*	3 (9%)
* ATRX*	1 (3%)
* BRCA*	1 (3%)
* FGFR*	1 (3%)
* FGFR-TACC*	1 (3%)
* IDH*	1 (3%)
* KIT*	1 (3%)
* TERT*	1 (3%)
* VEGFR*	1 (3%)
Proteins	
B7-H3	2 (6%)
MMP2	2 (6%)
CD147	1 (3%)
CND1/2	1 (3%)
mTOR	1 (3%)
p53	1 (3%)
PD-L1	1 (3%)
PDGFRa	1 (3%)
pERK	1 (3%)
Studies investigating systemic therapies (*n* = 48)^a^	
Targeted treatment	
Other	15 (31%)
Protein kinase inhibitor	8 (17%)
Tyrosine kinase inhibitor	8 (17%)
PARP inhibitor	5 (10%)
* EGFR* inhibitor	3 (6%)
Immunotherapy	
CAR-T	10 (21%)
Monoclonal antibody	6 (13%)
Other	4 (8%)
Other	
Acetazolamide	1 (2%)
Mycophenolate mofetil	1 (2%)

^a^Total number of therapies is 61 in these studies together. Chemotherapy in studies combining therapy with chemotherapy is not shown

### Targets and therapies investigated

Looking somewhat further into detail, *EGFR* (mutation or amplification**,** *n* = 11) was the most frequently investigated gene, followed by *CDKN2A/B or C* (deletion), *CDK4/6* (amplification), and *RB* (wild-type status), each being investigated in 4 studies*.* Of the protein targets, B7-H3 and MMP2 were the most frequently (*n* = 2 each) studied, both in the context of chimeric antigen receptor T-cell (CAR-T) therapy *(*Table 1*)*. All these alterations were used as a selection criterium for study participation.

Systemic therapies were investigated in 48 of the 56 studies on molecular criteria, but not all these studies required upfront matching based on at least one genomic alteration (Table 2). The majority (*n* = 27) of these therapeutic studies investigated one or more targeted therapies. Within the targeted therapy group, abemaciclib was the most frequently studied target-matched (*CDKN2A/B/C, CDK4/6, RB*) drug. Ribociclib, targeting the same genomic alterations, was the second most frequently studied drug. Focusing on immunotherapies, CAR-T therapies were the most frequently studied therapies that, inherently to the principle of CAR-T therapy, required upfront matching based on a genomic alteration. Other therapies being studied in recurrent glioblastoma included acetazolamide and mycophenolate mofetil, both known for potentiating chemosensitivity. In the study on acetazolamide, patients receive concomitant temozolomide, and Bcl-3 expression level is determined to examine the ability of Bcl-3 to predict treatment response. Mycophenolate is studied in combination with temozolomide and/or radiation therapy, and as an exploratory objective, molecular characterization of all glioblastoma tissues by RNAseq is performed.

**Table 2:** Systemic therapies currently being investigated in recurrent glioblastoma

Systemic therapy	Molecular matching criterium	ClinicalTrials.gov ID	Study phase
Targeted therapy			
Abemaciclib	*CDKN2A/B/C* inactivation or *CDK4/6* amplification and *RB* wild-type *CDKN2A/B/C* inactivation or *CDK4/6* amplification and *RB* wild-type *CDKN2A/B* inactivation or *CDK4/6* amplification	NCT02981940 NCT04391595 NCT04074785	Phase 2 Early phase 1 Early phase 1
Abexinostat	–	NCT05698524	Phase 1
Afatinib	*EGFR* amplification	NCT05432518	Early phase 1
Anlotinib	*VEGFR/PDGFR/FGFR/Kit* mutation (not specified)	NCT04004975	Phase 2
BDTX-1535	*EGFR* amplification/mutation/variant	NCT05256290	Phase 1–2
Bevacizumab	– – – – –	NCT05540275 NCT02974621 NCT03890952 NCT04074785 NCT02142803	Phase 2 Phase 2 Phase 2 Early phase 1 Phase 1
Cabozantinib	–	NCT05039281	Phase 1–2
Cediranib	–	NCT02974621	Phase 2
Cetuximab	*EGFR* overexpression	NCT02800486	Phase 2
CM93	*EGFR* mutation/amplification	NCT04933422	Phase 1
Dasatinib	*PDGFR* amplification	NCT05432518	Early phase 1
Everolimus	PI3K/*PTEN*/mTOR activated pathways	NCT05432518	Early phase 1
Lapatinib	*EGFR* amplification	NCT02101905	Phase 1
LY3214996	pERK positivity > 30%	NCT04391595	Early phase 1
Navtemadlin	p53 wild–type	NCT03107780	Phase 1
Niraparib	– *ATRX* loss	NCT05297864 NCT05076513	Phase 2 Early phase 1
Olaparib	*TP53* mutation –	NCT05432518 NCT02974621	Early phase 1 Phase 2
Osimertinib	*EGFR* amplification/mutation	NCT03732352	Phase 2
Palbociclib	*CDK4/6* amplification	NCT05432518	Early phase 1
Ribociclib	*RB* positivity *RB* wild-type and *CDKN2A/B/C* loss or *CDK4/6* amplification or CND1/2 amplification or 9p21.3 deletion	NCT02345824 NCT02933736	Phase 1 Early phase 1
Sapanisertib	– –	NCT02133183 NCT02142803	Phase 1 Phase 1
Selinexor	–	NCT05432804	Phase 1–2
Sorafenib	PDGFRa expression	NCT01817751	Phase 2
Talazoparib	*IDH* mutation*, PTEN* mutation*,* ‟*BRCA*ness” signature	NCT04740190	Phase 2
Temsirolimus	mTOR activation	NCT05773326	Early phase 1
Trastuzumab-deruxtecan	*HER2* expression	NCT06058988	Phase 2
Verteporfin	*EGFR* amplification/mutation	NCT04590664	Phase 1–2
Immunotherapy			
Anti-PD-L1 CSR T cells	PD-L1 positivity	NCT02937844	Phase 1
Atezolizumab	– –	NCT06069726 NCT05039281	Phase 2 Phase 1–2
CAR-T B7-H3	B7-H3 positivity B7-H3 positivity	NCT04385173 NCT04077866	Phase 1 Phase 1–2
CAR-T CD147	CD147 positivity	NCT04045847	Early phase 1
CAR-T Chlorotoxin	MMP2 + expression	NCT04214392	Phase 1
CAR-T CHM-1101	MMP2 + expression	NCT05627323	Phase 1
CAR-T EGFR-IL13Ra2 cells	*EGFR* amplification	NCT05168423	Phase 1
CAR-T EGFRvIII	EGFRvIII expression EGFRvIII expression EGFRvIII expression	NCT05802693 NCT02844062 NCT06186401	Early phase 1 Phase 1 Phase 1
CARv3-TEAM-E T cells	EGFRvIII mutation/*EGFR* amplification –	NCT05660369 NCT05024175	Phase 1 Phase 1
Erdafitinib	*FGFR–TACC* fusion	NCT05859334	Phase 2
Erlotinib	–	NCT00054496	Phase 2
Ezabenlimab	–	NCT03383978	Phase 1
Lerapolturev	–	NCT04479241	Phase 2
Memory-enriched T cells	*HER2* expression	NCT03389230	Phase 1
Nivolumab	–	NCT03890952	Phase 2
NK-92/5.28.z	*HER2* expression	NCT03383978	Phase 1
Pembrolizumab	– –	NCT04479241 NCT03277638	Phase 2 Phase 1–2
Tislelizumab	*PTEN/TERT* mutation (not specified)	NCT05540275	Phase 2
Other			
Acetazolamide	–	NCT03011671	Phase 1
Mycophenolate mofetil	–	NCT05236036	Phase 1

## Discussion

This study aimed to assess the current clinical trial landscape to assess the role of molecular biomarkers in trials on recurrent glioblastoma treatment. In 76% (181/237) of the included studies, molecular criteria (other than diagnostic) are not included in the study design. *EGFR* amplifications/ mutations are the most frequently investigated genomic alterations, followed by *CDKN2A/B* or* C* deletion*, CDK4/6* amplification, and *RB* wild-type status*.* Abemaciclib and ribociclib are the most frequently studied targeted therapies, while CAR-T therapies form the majority of our selection of the current trials on immunotherapy.

Currently, the established treatment options for patients with recurrent glioblastoma remain limited and far from being targeted to individual molecular characteristics [1]. Despite several attempts, the results of genome-driven oncology in the glioblastoma population so far are mixed and mostly disappointing [15]. First, the role of the blood–brain barrier and the blood–tumor barrier in relation to the efficacy of targeted treatments is an important factor to take into account.[7, 8] In addition, presence of a potential target does not automatically mean initiation of targeted treatment: an implementation gap is noticed between the finding of hypothetical druggable targets and the acting on that finding [16]. Challenges for genome-driven oncology as observed in that study include target credentialing and validation, tumor heterogeneity and clinical trial design. Notwithstanding these challenges, experts emphasize the need for (confirmatory) studies to further study the actual actionability of biomarkers in glioblastoma patients [1, 6]. An excellent example is the N2M2 study in patients with newly diagnosed glioblastoma without methylation of the O6-methylguanine-DNA methyltransferase (MGMT) promoter, a phase I/IIa umbrella trial of molecularly matched targeted therapies [17]. The recently presented results of this N2M2 study (NCT03158389) show clinical activity of temsirolimus in patients demonstrating mTOR activation, while palbociclib has no clinical activity in patients with *CDK4* amplification or *CDKN2A/B* codeletion.

Our assessment of the clinical trial landscape shows that the majority (76%) of the current trials aim to treat recurrent glioblastoma regardless the molecular characteristics of the tumor. More specifically, studies with upfront selection based on molecular alteration(s) to study the efficacy of certain drugs form a minority (14%) of the current clinical trial landscape. These early phase studies, in turn, are weakened by the fact that molecular testing on fresh tumor material at recurrence is required in less than 30 percent of the studies. Reflecting on these outcomes, some comments need to be made. First of all, the yield of extensive molecular screening for potentially actionable alterations and subsequent targeted treatment is not undebated. For instance, after NGS analysis in more than 400 glioblastoma patients, personalized treatment was initiated in only 11% of the patients [18]. At the same time, WGS analyses showed that glioblastomas harbor potentially actionable alterations in the majority of the cases [19, 20]. A second remark is that trials with extensive molecularly analyzed glioblastomas require good access to molecular tests, which is not the case all over the world. Third, the observation that fresh tumor material at recurrence is not required in the majority of the studies, which may be indicative of the fact that current standard practices prove difficult to adapt to optimal molecular diagnostics.

This study has some limitations to be considered. First, the selection of the clinical trials was purely based on the registration on ClinicalTrials.gov, which allows for an incomplete snapshot of the trials going on since new studies can be registered on ClinicalTrials.gov on a daily basis. A second limitation is that the recruitment status of a study could be outdated since actual status is dependent on update information provided by the research team itself. As a result, studies no longer recruiting may have been erroneously included in this assessment of the current trial landscape. On the other hand, our study design ruled out studies no longer recruiting, potentially resulting in the loss of interesting new information on treatment targets. Nevertheless, the methods used in this assessment ensure a fair assessment and indication of the current clinical trial landscape. Finally, this study did not investigate (recently) completed or terminated trials, which would have been interesting to compare previously studied targeted drugs with currently experimental therapies. As a result, our study does not allow any conclusions about past efforts in the field of genome-driven oncology for patients with recurrent glioblastoma.

To conclude, this study provided an insight into the current trials on the role of molecular biomarkers in trials on recurrent glioblastoma treatment. Currently, the need for new studies with upfront selection based on molecular alteration(s) to study the efficacy of certain drugs is not yet translated into genome-driven trials being conducted. Our results emphasize that, in order to move the field of neuro-oncology into the direction of personalized medicine and to bridge the knowledge gap, an intensification of genome-driven trials is needed.

## Data Availability

The datasets used and/or analyzed during the current study available from the corresponding author on reasonable request.
